# The Neuropeptide 26RFa (QRFP) and Its Role in the Regulation of Energy Homeostasis: A Mini-Review

**DOI:** 10.3389/fnins.2016.00549

**Published:** 2016-11-29

**Authors:** Nicolas Chartrel, Marie Picot, Mouna El Medhi, Arnaud Arabo, Hind Berrahmoune, David Alexandre, Julie Maucotel, Youssef Anouar, Gaëtan Prévost

**Affiliations:** ^1^INSERM U982, Laboratory of Neuronal and Neuroendocrine Differentiation and Communication, Institute for Research and Innovation in Biomedicine, University of Rouen, Normandy UniversityMont-Saint-Aignan, France; ^2^University of Rouen, Normandy UniversityMont-Saint-Aignan, France; ^3^Department of Endocrinology, Diabetes and Metabolic Diseases, Institute for Research and Innovation in Biomedecine, University Hospital of Rouen, University of Rouen, Normandy UniversityRouen, France

**Keywords:** RFamide peptide, G protein-coupled receptor, food intake, glucose homeostasis, obesity, diabetes

## Abstract

This mini-review deals with the neuropeptide 26RFa (or QRFP) which is a member of the RFamide peptide family discovered simultaneously by three groups in 2003. 26RFa (or its N-extended form 43RFa) was subsequently shown to be the endogenous ligand of the human orphan receptor GPR103. In the brain, 26RFa and GPR103mRNA are primarily expressed in hypothalamic nuclei involved in the control of feeding behavior, and at the periphery, the neuropeptide and its receptor are present in abundance in the gut and the pancreatic islets, suggesting that 26RFa is involved in the regulation of energy metabolism. Indeed, 26RFa stimulates food intake when injected centrally, and its orexigenic effect is even more pronounced in obese animals. The expression of 26RFa is up-regulated in the hypothalamus of obese animals, supporting that the 26RFa/GPR103 system may play a role in the development and/or maintenance of the obese status. Recent data indicate that 26RFa is also involved in the regulation of glucose homeostasis. 26RFa reduces glucose-induced hyperglycemia, increases insulin sensitivity and insulinemia. Furthermore, an oral ingestion of glucose strongly stimulates 26RFa release by the gut, indicating that 26RFa is a novel incretin. Finally, 26RFa is able to prevent pancreatic β cell death and apoptosis. This brief overview reveals that 26RFa is a key neuropeptide in the regulation of energy metabolism. Further fields of research are suggested including the pathophysiological implication of the 26RFa/GPR103 system.

## Discovery of 26RFa and its receptor GPR103

26RFa also referred to as QRFP (for pyroglutamilated RFamide peptide) is a 26-amino acid peptide discovered simultaneously by three different groups in 2003 including our team (Chartrel et al., 2003; Fukusumi et al., 2003; Jiang et al., 2003). These teams have developed either a bioinformatic approach or a comparative approach using frog brains as a source of neuropeptides (Chartrel et al., 2002, 2003; Fukusumi et al., 2003; Jiang et al., 2003). 26RFa and/or its N-terminal extended form, 43RFa, have been subsequently biochemically characterized in the human hypothalamus and the rat brain (Bruzzone et al., 2006; Takayasu et al., 2006), and molecular cloning has revealed that a 26RFa precursor-encoding sequence is present in human (Chartrel et al., 2003; Fukusumi et al., 2003), ox (Fukusumi et al., 2003), rat (Chartrel et al., 2003; Fukusumi et al., 2003; Jiang et al., 2003), mouse (Fukusumi et al., 2003; Jiang et al., 2003), quail (Ukena et al., 2010), chicken (Ukena et al., 2010), and goldfish (Liu et al., 2009), indicating that 26RFa is widely distributed among vertebrates.

26RFa is the cognate ligand of the human orphan receptor GPR103, also designated SP9155 or AQ27 (Chartrel et al., 2003, 2011; Jiang et al., 2003). GPR103 is a 7-transmembrane G protein-coupled receptor (GPCR) that shares significant amino acid identity (52%) with NPFF2 (Bonini et al., 2000; Lee et al., 2001), another receptor for mammalian RFamide peptides. Binding studies and functional assays indicated that the N-elongated form of 26RFa, 43RFa, binds also with a high affinity to GPR103 and has the same efficacy as 26RFa to inhibit cAMP production (Fukusumi et al., 2003; Jiang et al., 2003). In contrast, GPR103 is not activated by other mammalian RFamide peptides, such as PrRP, RFRP-1 and -3 (Dockray, 2004), indicating that GPR103 selectively recognizes 26RFa/43RFa. Conversely, 26RFa displays moderate affinity and selectivity for NPFF-2 (Gouardères et al., 2007). Data mining revealed that two orthologues of human GPR103 are present in the mouse and rat genome (Kampe et al., 2006; Takayasu et al., 2006). The two GPR103 genes from rodents exhibit between 79 and 85% homology with human GPR103 (Kampe et al., 2006; Takayasu et al., 2006). 26RFa/43RFa bind with a similar affinity the two forms of GPR103 in both mouse and rat (Kampe et al., 2006; Takayasu et al., 2006). Up to now, the occurrence of two distinct GPR103 receptors has only been reported in rodents.

## 26RFa and control of feeding behavior

Neuroanatomical studies have revealed a discrete localization of 26RFa-expressing neurons in various hypothalamic nuclei including the ventromedial hypothalamic nucleus (VMH), the lateral hypothalamic area (LHA) and the arcuate nucleus (Arc) (Chartrel et al., 2003; Bruzzone et al., 2007). GPR103-containing neurons are found in the same hypothalamic structures but also in other brain nuclei, such as the piriform cortex and the nucleus of the solitary tract (Bruzzone et al., 2007). All of these nuclei mentioned above are known to be involved in the control feeding behavior raising the hypothesis that 26RFa may be implicated in the hypothalamic regulation of food intake. This is the case as intracerebroventricular (i.c.v.) administration of 26RFa in mice stimulates food consumption in a dose-dependent manner, and expression of the 26RFa precursor is up-regulated in the hypothalamus of fasted mice (Chartrel et al., 2003; Do Rego et al., 2006; Takayasu et al., 2006). 43RFa exerts a similar effect and is even more potent than 26RFa in stimulating appetite (Do Rego et al., 2006; Moriya et al., 2006; Takayasu et al., 2006). In addition, chronic administration of 43RFa for 2 weeks results in an important increase of body weight and fat mass in mice that also exhibit a hyperphagic behavior (Moriya et al., 2006). These effects of 43RFa are more pronounced when mice are fed a moderately high fat diet (Moriya et al., 2006). Finally, 26RFa mRNAs are increased in genetically obese *ob/ob* and *db/db* mice (Takayasu et al., 2006), suggesting that up-regulation of 26RFa may play an important role in the maintenance of obesity.

26RFa has also been found to stimulate food intake in rats fed a standard chow (Kampe et al., 2006; Lectez et al., 2009). Consistent with this observation, it has been recently shown that direct administration of 26RFa into the medial hypothalamus increases food consumption (Zagorácz et al., 2015), and that the concentrations of 26RFa/43RFa in the VMH are significantly increased in rats fed a standard chow (Beck and Richy, 2009). It has also been found that 26RFa still stimulates appetite when rats are fed a high fat diet (Primeaux et al., 2008), and this phenomenon is accompanied by an up-regulation of prepro26RFa and GPR103 in the VMH and the Arc (Schreiber et al., 2016). By contrast, these authors (Primeaux et al., 2008; Schreiber et al., 2016) as well as Patel et al. (2008) failed to find any effect of 26RFa or 43RFa on food consumption when rats are fed a standard chow. Interestingly, in both mice and rats, 26RFa potently stimulates food intake when the animals are deprived of food for 18 h prior to the injection of the neuropeptide (Chartrel et al., 2003; Do Rego et al., 2006; Lectez et al., 2009) strongly suggesting that starvation potentiates the orexigenic activity of 26RFa. To conclude, these data indicate that in both mice and rats, 26RFa/43RFa strongly stimulate food consumption when the animals are fed a moderate or a high fat diet (Moriya et al., 2006; Primeaux et al., 2008), and that the expression of prepro26RFa is enhanced in the hypothalamus of animals submitted to such a fat diet (Moriya et al., 2006; Primeaux et al., 2008). These data support therefore the notion that 26RFa/43RFa plays a role in the establishment and maintenance of the obese status in mammals. However, Beck and Richy (2009) have recently reported a decrease of 43RFa levels in the VMH of rats fed a high fat diet. Conversely, a single study has investigated the expression/production of 26RFa under chronic undernutrition (Galusca et al., 2012). This study has been conducted in young women suffering from anorexia nervosa in which circadian plasma 26RFa levels have been measured. The data reveal significant higher levels of circulating 26RFa in anorectic patients as compared to healthy volunteers, suggesting the occurrence of an adaptive mechanism of the organism to promote energy intake and to increase fat stores in response to chronic undernutrition (Galusca et al., 2012).

Interestingly, it has been reported that 26RFa promotes arousal in mice (Takayasu et al., 2006), raising the hypothesis that the orexigenic activity of the neuropeptide may be related to its wake-promoting effect, as previously suggested for the other orexigenic neuropeptide orexin. However, a recent paper reveals that, in the zebrafish, the overexpression of 26RFa in the hypothalamus inhibits locomotor activity and promotes sleep whereas lack of 26RFa signaling results in increased locomotor activity and decreased sleep during the day (Chen et al., 2016).

One neuronal pathway by which 26RFa/43RFa exerts its orexigenic activity in the hypothalamus has been elucidated. The investigation has focused on the neuropeptide Y (NPY)/proopiomelanocortin (POMC) system of the Arc as a high expression of the 26RFa receptor is found in this nucleus (Sakurai et al., 1998; Fukusumi et al., 2003; Takayasu et al., 2006; Bruzzone et al., 2007). i.c.v. administration of 26RFa induces an increase in the expression and release of NPY in the Arc and, simultaneously, a decrease in POMC expression and α-MSH release (an anorexigenic POMC-derived peptide) which is associated with an increase in food consumption (Lectez et al., 2009). In addition, in this study, Lectez et al. (2009) show that the effects of 26RFa on the activity of POMC neurons is indirect as these neurons do not express the GPR103 transcript. Specific antagonists of the Y1 and Y5 NPY receptors (which are expressed by POMC neurons) totally abolish the inhibitory effect of 26RFa on POMC expression and α-MSH release, as well as 26RFa-induced food intake, indicating that 26RFa increases consumption by stimulating the release of NPY which in turn inhibits the activity of POMC neurons via the activation of the Y1 and Y5 receptors (Lectez et al., 2009, Figure 1). A possible involvement of the orexin system in the orexigenic hypothalamic effect of 26RFa has also been examined because the orexin system shows important similarity with the 26RFa/GPR103 system. As a matter of fact, orexins stimulate food intake and orexin-expressing neurons are localized in the LHA (Sakurai et al., 1998), like 26RFa. The orexin neurons innervate the NPY neurons of the Arc (Ciriello et al., 2003) that express the OX-R1 receptor (Bäckberg et al., 2002). Orexin-A stimulates the activity of NPY neurons (López et al., 2002) and the orexigenic activity of the neuropeptide is abolished when the animals have previously received Y1 and Y5 NPY receptor antagonists (Dube et al., 2000; Yamanaka et al., 2000). Finally, it has been recently demonstrated that orexin receptors and GPR103 can form functional heterodimers to exert their effects through activation of ERK (Davies et al., 2015). However, the fact that 26RFa still stimulates appetite in orexin invalidated mice (Takayasu et al., 2006) indicates that the orexin system is not recruited during 26RFa-induced food intake. Alternatively, it is not known whether the OX receptor needs to dimerise with GPR103 to initiate orexin-induced food intake or whether orexin and 26RFa may act synergically via heterodimerization of their receptors to potentiate their orexigenic activities. This interesting hypothesis deserves further investigation.

**Figure 1:**
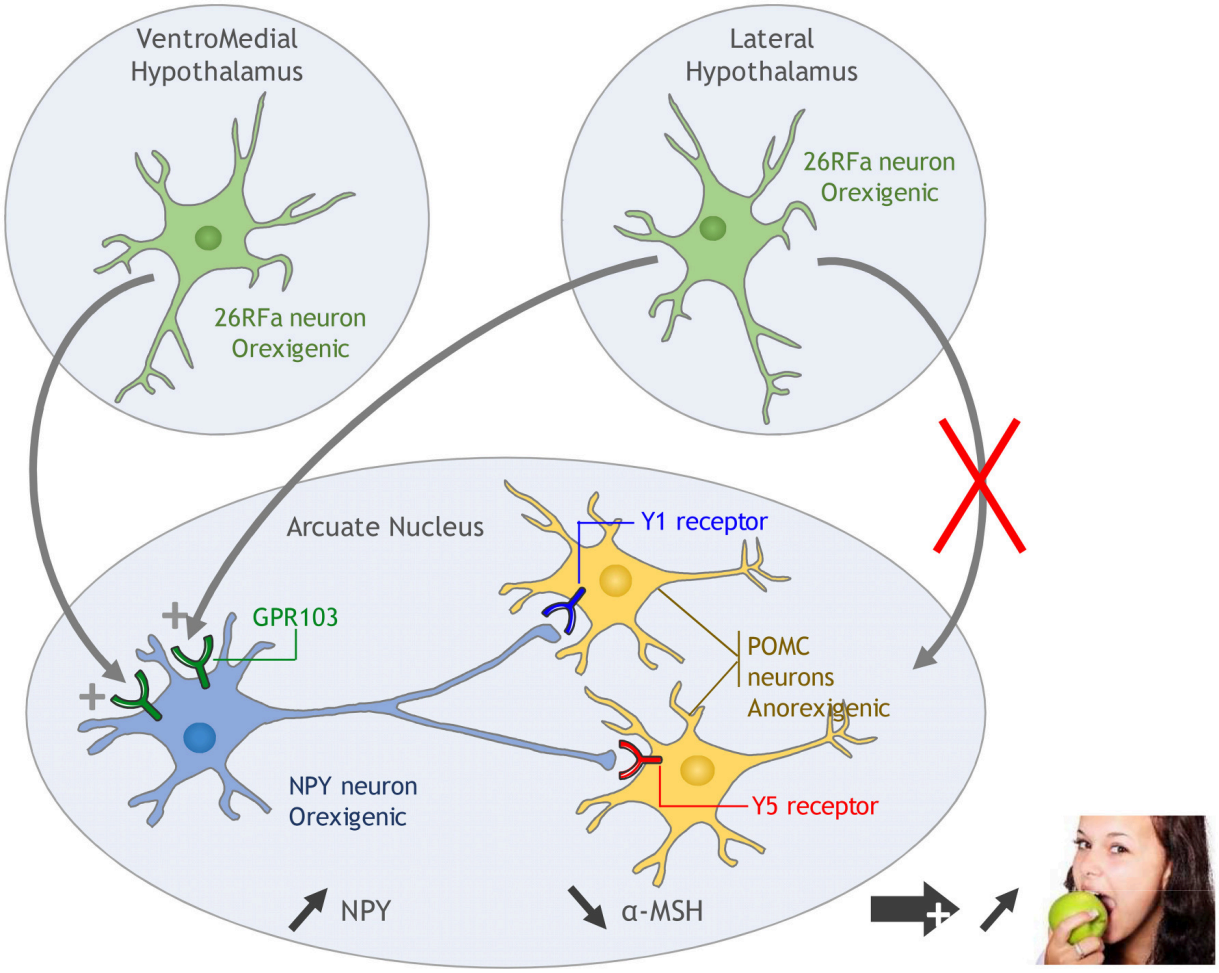
**Proposed mechanism of action of 26RFa in the hypothalamic control of food intake**. 26RFa, produced by neurons of the ventromedial hypothalamic nucleus (VMH) and the lateral hypothalamic area (LHA), stimulates the activity of NPY neurons of the arcuate nucleus (Arc) via activation of GPR103. Subsequent NPY release in the Arc inhibits the activity of proopiomelanocortin (POMC) neurons via activation of the Y1 and Y5 receptors, leading to a stimulation of appetite.

In addition to its central action, accumulating data indicate that 26RFa can also regulate energy homeostasis at the periphery. It has notably been shown that, in the adipocyte cells 3T3-L1, 26RFa, and 43RFa stimulate triglyceride accumulation and fatty acid uptake, and increase the expression of genes involved in lipid uptake (Mulumba et al., 2010). Concurrently, the expression of GPR103 is enhanced in the adipose tissue of a mouse model of diet-induced obesity whereas that of prepro26RFa is decreased, and the neuropeptide inhibits lipolysis in adipocytes of these animals (Mulumba et al., 2010, 2015). It thus appears that 26RFa plays a crucial role in the central and peripheral regulation of body weight and energy homeostasis, and may be involved in the development and maintenance of obesity in vertebrates.

## 26RFa and control of glucose homeostasis

Type 2 diabetes, which is a frequent consequence of obesity, is characterized by chronic hyperglycemia induced by impaired insulin secretion due to decreased β cell mass and function, and increased insulin resistance (Butler et al., 2003; Kahn et al., 2014). Recent studies suggest a peripheral role of hypothalamic neuropeptides controlling feeding behavior in the regulation of glucose homeostasis, leading to the new concept that hypothalamic neuropeptides may serve as a link between energy and glucose homeostasis, and identifying them therefore as potential therapeutic targets for the treatment of diabetes and obesity (Greenwood et al., 2011). With regard to these observations a potential role of 26RFa/43RFa in the regulation of glucose homeostasis has been examined. A primary study in 2007 has investigated the effect of 26RFa on insulin and glucagon secretion by rat perfused pancreas (Egido et al., 2007). This study reports that 26RFa reduces glucose, arginine and exendin-4 (a GLP-1 agonist)-induced insulin release without affecting glucagon secretion (Egido et al., 2007). In addition, these authors show that the inhibitory effect of 26RFa on exendin-4-induced insulin release is not observed in pancreas from pertussis toxin-treated rats suggesting the involvement of a pertussis toxin-sensitive G_i_ protein negatively coupled to the adenylyl cyclase system (Egido et al., 2007). Recently, the role and mechanism of action of 26RFa/43RFa in the regulation of glucose metabolism has been studied more thoroughly (Granata et al., 2014; Prévost et al., 2015). The two studies show that 26RFa/43RFa and GPR103 are expressed by the pancreatic islets as well as by the rodent insulin-secreting cell lines INS-1E and MIN6. Granata et al. (2014) report that 26RFa and 43RFa prevent cell death and apoptosis induced by serum starvation, cytokines and glucolipotoxicity in INS-1E β cells and in isolated human pancreatic islets. In addition, these authors indicate that 43RFa promotes, whereas 26RFa inhibits, glucose—and exendin-4-induced insulin secretion through Gα_s_ and Gα_i/o_ proteins, respectively (Granata et al., 2014). They also show that inhibition of GPR103 expression by small interfering RNA in INS-1E β cells totally blocks the insulinotropic effect of 43RFa but not the insulinostatic action of 26RFa, suggesting that the insulinotropic effect of 43RFa is mediated via activation of GPR103 whereas, conversely, the insulinostatic effect of 26RFa is mediated via another unknown receptor (Granata et al., 2014). Finally, the same study reveals that 43RFa promotes glucose uptake by β cells whereas 26RFa does not (Granata et al., 2014).

Our team has also investigated the role and mechanism of action of 26RFa in the regulation of glucose homeostasis (Prévost et al., 2015). Clinical studies performed in human revealed a positive correlation between plasma 26RFa and plasma insulin in obese, type 2 diabetic patients and healthy volunteers. In addition, measurement of plasma 26RFa during an oral glucose tolerance test shows an increase in the circulating levels of the neuropeptide during the test, indicating a link between the 26RFa system and glucose homeostasis (Prévost et al., 2015). Finally, immunohistochemical experiments describe the presence, in abundance, of 26RFa in the gut, from the stomach to the colon, suggesting that the gut is the primary source of circulating 26RFa that can be released under an oral glucose load. In mice, it was found that i.p. administration of 26RFa does not alter basal glycemia. In contrast, the neuropeptide strongly attenuates glucose-induced hyperglycemia during a glucose tolerance test, indicating that the neuropeptide exerts an antihyperglycemic effect rather than a hypoglycemic effect. In addition, it is reported that 26RFa enhances insulin sensitivity and increases insulin production, suggesting that the two mechanisms contribute to the antihyperglycemic effect of 26RFa. 26RFa-induced insulin production is due to a direct action of the neuropeptide on pancreatic β cells as 26RFa stimulates insulin release by the MIN6 cells that express GPR103, and as invalidation of GPR103 in these cells totally abolishes 26RFa-induced insulin secretion (Prévost et al., 2015). These observations are partially in agreement with those of Granata et al. (2014) reporting that 43RFa stimulates insulin secretion by human pancreatic islets and INS-1E β cells by activating GPR103. However, the same authors found that 26RFa exerts an opposite effect to that of 43RFa, and that the inhibiting effect of 26RFa on insulin secretion is not mediated by GPR103. They thus suggest that NPFF2, another receptor that 26RFa can bind to but with a much lower affinity than to GPR103 (Moriya et al., 2006), may be involved in the insulinostatic activity of 26RFa. In our study (Prévost et al., 2015), we show that MIN6 cells do not express NPFF2 that might explain the discrepancy between the two studies.

**Figure 2:**
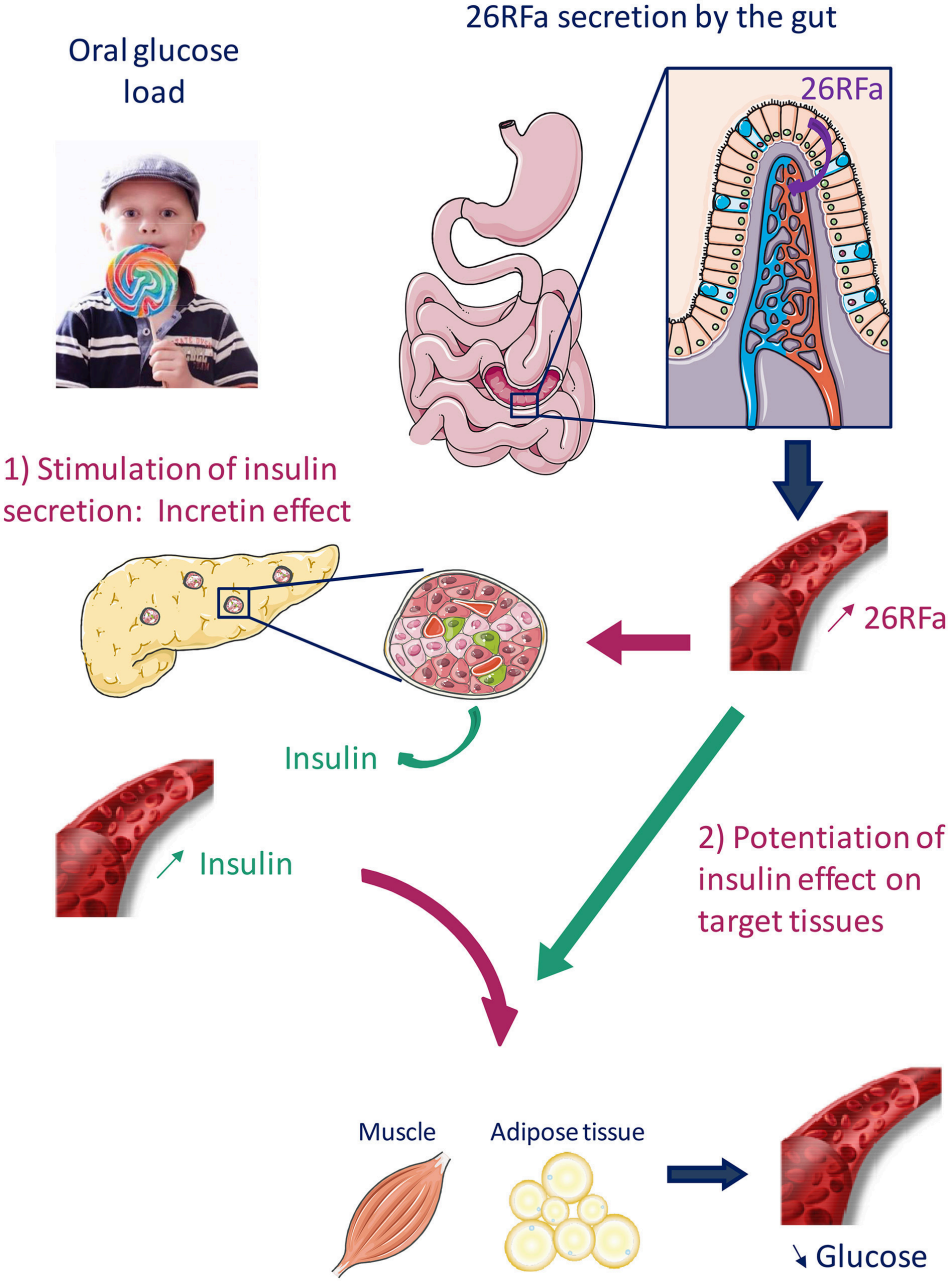
**Proposed mechanism of action of 26RFa in the control of glucose homeostasis**. 26RFa is abundantly produced by the gut and released in the general circulation after an oral glucose load. 26RFa stimulates insulin release by pancreatic cells and potentiates insulin sensitivity on target tissues (muscle, adipose tissue), leading to a decrease of glycemia.

26RFa has also been found to increase insulin sensitivity and GPR103 is co-expressed with the insulin receptor and the glucose transporter, GLUT-4, in the muscle, liver and adipose tissue (Prévost et al., 2015). In addition, a recent study indicates that 26RFa enhances insulin's effects on glucose uptake in rat skeletal muscle cells (Allerton and Primeaux, 2015). Altogether, these observations strongly suggest a direct action of the neuropeptide on insulin target tissues. Finally, it has been observed that an oral ingestion of glucose induces an increase in plasma 26RFa levels 30 min after the glucose load, whereas this phenomenon is not observed when glucose is administrated i.v., indicating therefore that elevation of 26RFa in the blood is due to a massive release of the neuropeptide by the gut (Prévost et al., 2015). In conclusion, these studies promote evidence for an important role of 26RFa, acting as an incretin, in the regulation of glucose homeostasis (Figure 2). Interestingly, a genome wide identification of new genes causing type 1 diabetes with autoimmune thyroiditis has revealed a strong association of the GPR103 gene with this pathology (Tomer et al., 2015), supporting the involvement of the 26RFa/GPR103 system in the regulation of glucose metabolism.

## Conclusion

Accumulating data obtained during the last decade reveal that the 26RFa/GPR103 system plays an important role in the regulation of food intake and glucose homeostasis. Obesity associated with type 2 diabetes is a major world-wide problem of public health as 11% of the world population is obese (Organisation Mondiale de la Santé, 2016) and 400 millions people are affected by type 2 diabetes in the world (Kahn et al., 2014). One main axis for further research would be to investigate whether dysfunction of the 26RFa/GPR103 system is associated with diabetes/obesity that could serve as a basis to develop 26RFa analogs to treat the pathology. Supporting this idea, it can be reminded that, during the last decade, the therapeutic arsenal to treat type 2 diabetes has been enlarged with the occurrence of novel classes of drugs, such as GLP-1 agonists and inhibitors of DPPIV.

Besides, an increasing body of evidence supports the existence in the hypothalamus of a glucoregulatory system that acts coordinately with pancreatic islets to regulate blood glucose levels, via both insulin-dependent and insulin-independent mechanisms, that would be responsible of 50% of glucose homeostasis. Considering this latter observation, it would be important to investigate whether the hypothalamic neuronal populations expressing 26RFa are involved in the central regulation of glucose homeostasis.

## Author contributions

All of the authors have contributed to the design, writing and correction of the present mini-review.

## Funding

The work supported by the “Institut National de la Santé et de la Recherche Médicale” (INSERM U982) et the “Fondation pour la Recherche Médicale.”

### Conflict of interest statement

The authors declare that the research was conducted in the absence of any commercial or financial relationships that could be construed as a potential conflict of interest. The reviewer VT declared a shared affiliation, though no other collaboration, with several of the authors [NC; MP; ME; HB; DA; YA; GP] to the handling Editor, who ensured that the process nevertheless met the standards of a fair and objective review.
